# Lack of association or interactions between the IL-4, IL-4Rα and IL-13 genes, and rheumatoid arthritis

**DOI:** 10.1186/ar2454

**Published:** 2008-07-14

**Authors:** Ioanna Marinou, Simon H Till, David J Moore, Anthony G Wilson

**Affiliations:** 1 Section of Musculoskeletal Sciences, School of Medicine & Biomedical Sciences, The University of Sheffield, Royal Hallamshire Hospital, Beech Hill road, Sheffield S10 2RX, UK

## Abstract

**Introduction:**

A feature of rheumatoid arthritis (RA) is an imbalance between proinflammatory and anti-inflammatory cytokines. Several recent studies have implicated polymorphism in the IL-4 signalling pathway in the development of erosive RA. The aim of the present study was to investigate the role of polymorphism in the IL-4, IL-4Rα and IL-13 genes in RA, including an examination of epistasis.

**Methods:**

A total of 965 Caucasian patients with RA (cases) and 988 healthy control individuals (controls) were genotyped for five variants in the IL-4/IL-13 gene cluster (5q31.1) and two functional variants IL-4Rα (16p12.1). Individual genotype and haplotype frequencies were compared between cases and controls. The odd ratios were calculated with asymptotic 95% confidence intervals, and *P *values less than 0.05 were considered statistically significant. The potential association with radiological joint damage was also examined. Potential gene interactions were assessed using both stratified analysis and the linkage disequilibrium-based statistic.

**Results:**

Genotype, allele and haplotype frequencies were equally distributed between RA cases and controls. Similarly, no association was detected between these variants and modified Larsen scores. Furthermore, no evidence of epistasis was detected between IL-4 or IL-13 genotypes and IL-4Rα.

**Conclusion:**

These results indicate that common variants of the IL-4/IL-13 pathway do not significantly contribute to RA susceptibility and radiological severity.

## Introduction

Rheumatoid arthritis (RA) is the commonest autoimmune disease with a prevalence of 1% worldwide. Two loci, DRB1 and PTPN22, have been reproducibly implicated in the genetic background of RA. Advances in high-throughput genotyping and bioinformatics have allowed genome-wide assocation studies to be performed. The Wellcome Trust Case Control Consortium reported associations of nine novel variants with RA [1]. A subsequent UK case-control study confirmed association of one of the markers, rs6920220, with an intergenic region between the oligodendrocyte lineage transcription factor 3 and tumour necrosis factor (TNF)-α-induced protein 3 genes at 6q23.3 with RA [2]. However, of the other eight markers, only rs743777 in the *IL2RB *gene exhibited weak evidence of association. The association of the region encompassing *OLIG3-TNFAIP3 *was independently identified in a US study that combined both a case-control and genome-wide association study [3]. Another genome wide study involving 1,622 RA patients and 1,850 healthy control individuals revealed significant association within a 100 kilobase region on chromosome 9 encompassing the *TRAF1-C5 *genes [4]. The signal transducer and activator of transcription (STAT)4 gene was also recently implicated in susceptibility to RA [5].

A central feature of RA is a relative imbalance in proinflammatory and anti-inflammatory cytokines, with high levels of TNF, IL-1 and IL-6, but much lower levels of IL-4 and IL-13 [6]. The recognition that dysregulated production of cytokines was important in the pathogenesis of RA leads directly to the successful use of TNF and IL-1 inhibitors.

The genes for the anti-inflammatory cytokines IL-4 and IL-13 are located in a gene cluster at 5q31.1. Their effects are mediated by a heterodimeric receptor composed of the IL-4Rα chain (16p12.1) and either the common γ chain or the IL-13Rα subunit [5]. IL-4, also known as B-cell-stimulating factor, was the first B-cell growth factor discovered. It mainly promotes proliferation of T cells and induces antibody production by B cells. IL-13 is a T-cell-specific cytokine that has a 30% protein homology with IL-4, and it stimulates proliferation and differentiation of B cells and induces IgE and IgM production.

The role played by these genes in RA susceptibility and severity is not clearly established. An IL-4 variable number tandem repeat has been associated with both RA susceptibility and lesser radiological damage [7]. A promoter single nucleotide polymorphism (SNP) in IL-4 -590 (rs2243250), which is associated with enhanced IL-4 activity [8], has also been associated with more severe RA [9]. However, this polymorphism was not associated with RA susceptibility in a large case-control study [10]. The IL-4 -590 T allele is associated with threefold higher promoter activity as well as IgE production in Jurkat cells [11]. More recently, a study suggested that a nonsynonymous SNP located in the IL-4 binding site of the IL-4R gene was associated with rapidly erosive RA during the first 2 years of disease, and that this was independent of other risk markers such as HLA-DRB1 and autoantibodies [12].

In this study we examined the potential role of IL-4, IL-13 and IL-4R SNPs in RA susceptibility and severity. In addition, we also investigated epistasis between the IL-4 or IL-13 and their common receptor IL-4Rα.

## Materials and methods

### Study populations

A total of 965 white Caucasian individuals with RA (cases) and 988 healthy unrelated individuals (controls) participated in this study and were described previously [13]. The South Sheffield Research Ethics Committee approved this study and informed consent was obtained from all participants. RA was diagnosed according to the American College of Rheumatology diagnostic criteria. Radiographs of hands and feet were scored blind at study entry by a single musculoskeletal radiologist using a modification to Larsen's score [14]. Larsen's method measures the amount of destruction by assessing 32 joints, including 16 areas in both hands (eight joints in each hand), eight areas in both wrists (four joints in each wrist) and eight joints in both feet (four areas in each foot) [15].

### SNP selection

In addition to the functional SNPs I50V and Q551R in the IL-4R gene, SNPs in the IL-4 and IL-13 genes were selected using a haplotype-tagging approach. Haplotype-tagging SNPs were selected using the genotyping data from the CEU (Centre d'Etude du Polymorphisme Human from Utah) study group (30 US trios recruited in 1980 from US residents with Northern and Western European ancestry), available from the International Hapmap project [16], using the Tagger algorithm implemented by Paul de Bakker (available through Haploview software) [5]. Selection was undertaken using the pairwise-tagging option with an *r*^2 ^of 0.8 and minor allele frequency greater than 10%. We selected three IL-4 SNPs that allowed us to define the haplotypes described in the Hapmap project. Tagging of the promoter IL-4 -590 variant was achieved using rs2243267 (*r*^2 ^= 0.94). For IL-13 one SNP was selected capturing five additional SNPs. We also genotyped the promoter polymorphism -1112 (rs1800925), which has been shown to alter binding of the STAT transcription factor and levels of IL-13 in T cells [7].

### SNP genotyping

Blood samples were collected in EDTA-anticoagulated tubes and DNA was extracted using standard methods. All SNPs genotyped using Taqman technology (Applied Biosciences, ABI, Warrington, UK). Multiple positive and negative controls were included in all genotyping plates to ensure genotyping data and 10% of the samples were repeated to eliminate genotyping errors. Multiple positive and negative controls were included in all genotyping plates to ensure quality of genotyping data. Genotypes were also confirmed by sequencing. Thermal cycling in 384-well plates was performed on PTC-225 DNA engine Tetrad (MJ Research, San Francisco, CA, USA), and genotypes were determined using an ABI Prism 7900 HT (PE Biosystems, Foster City, CA, USA).

### Statistical analysis

Hardy-Weinberg equilibrium (HWE) of each SNP was assessed in cases and controls separately using a χ^2 ^test with one degree of freedom. A threshold *P *< 0.05 was regarded to indicate deviation from HWE. Allele and genotype frequencies were calculated and associations with susceptibility to RA were tested by calculating odds ratios (ORs) with asymptotic 95% confidence intervals (CIs), and *P *values less than 0.05 were considered statstically significant. Haplotype association analysis was performed using SNPStats, a web-based tool that allows the estimation of haplotypes frequencies using the expectation-maximization (EM) algorithm.

Gene-gene interactions between different variants were tested using two different approaches. We stratified the study subjects according to the genotype of one gene and performed the analysis of the other gene on different strata using the Mantel-Haenszel test. Deviation from independence of penetrance in two unlinked loci (referred to as the linkage disequilibrium [LD]-based statistic) was also used to detect gene-gene interactions; this method is derived from the assumption that interaction between two unlinked loci creates LD in a disease population, the level of which depends on the extent of the interaction. A total of seven tests for main effects and 10 for gene-gene interactions (17 tests in total) were performed. All statistical analyses were carried out using STATA statistical software (Release 9.1; STATA Corporation, College Station, TX, USA).

We then examined the potential influence of each variant in disease severity. Dichotomized variables were created for both rheumatoid factor and anti-cyclic citrullinated peptide levels, as previously described. Modified Larsen score differences were tested for associations with each candidate gene polymorphism using the nonparametric Kruskal-Wallis test.

## Results

### Single marker association analysis of IL-4, IL-13 and IL-4R SNPs with RA susceptibility

We successfully genotyped SNPs in IL-4, IL-13 and IL-4R genes, tagging one block for IL-4 and one block for IL-13. All polymorphisms under study were in HWE in both cases and controls, separately. Tagging of the promoter IL-4 -590 variant was achieved using rs2243267. No significant associations were detected (Table 1) apart from a marginal association of the IL-4R AG (I50/V50) genotype (50.7% versus 46.6%; OR = 1.3, 95% CI = 1.0 to 1.6; *P *= 0.03). No other association was detected in the single SNP analysis.

**Table 1:** Genotype frequencies and RA susceptibility

Gene	SNP	Genotypes	RA (*n *[%])	Controls (*n *[%])	OR (95% CI)	*P *value
IL-13	rs1800925	TT	26 (2.9)	26 (2.8)	1.0 (0.6–1.9)	0.9
		CT	271 (29.9)	276 (29.6)	1.0 (0.8–1.3)	0.9
		CC	608 (67.2)	631 (67.6)	1.0	
	rs1295686	TT	26 (3.0)	31 (3.3)	0.9 (0.5–1.6)	0.7
		CT	251 (29.3)	283 (30.4)	0.9 (0.8–1.2)	0.6
		CC	580 (67.7)	617 (66.3)	1.0	
IL-4	rs2227284	AA	56 (6.1)	47 (4.9)	1.2 (0.8–1.9)	0.3
		AC	316 (34.6)	349 (36.7)	0.9 (0.8–1.1)	0.4
		CC	541 (59.3)	554 (58.3)	1.0	
	rs2243263	CC	8 (0.9)	6 (0.6)	1.4 (0.4–4.8)	0.6^a^
		GC	146 (16.4)	166 (17.9)	0.9 (0.7–1.2)	0.4
		GG	738 (82.7)	753 (81.4)	1.0	
	rs2243267	CC	18 (2.0)	17 (1.8)	1.1 (0.5–2.3)	0.8^a^
		CG	218 (24.0)	205 (22.2)	1.1 (0.9–1.4)	0.4
		GG	672 (74.0)	702 (76.0)	1.0	
IL-4R	rs1805010	GG	198 (21.8)	200 (21.3)	1.2 (0.9–1.5)	0.2
		AG	460 (50.7)	438 (46.6)	1.3 (1.0–1.6)	0.03
		AA	250 (27.5)	301 (32.1)	1.0	
	rs1801275	AA	38 (4.2)	41 (4.3)	1.0 (0.6–1.6)	1.0
		AG	316 (34.6)	302 (32.0)	1.1 (0.9–1.4)	0.2
		GG	558 (61.2)	601 (63.7)	1.0	

^a^Fisher's exact test. CI, confidence interval; OR, odds ratio; RA, rheumatoid arthritis.

### LD and haplotype analysis

To assess the extent of LD across the IL-4 and IL-13 genes, we calculated D' values. The pair-wise D' values in the IL-4 gene were nearly 1 (Table 2) among all SNPs, indicating very strong LD. Interestingly, the IL-13 SNPs were not in strong LD with each other or with the IL-4 polymorphisms (D' < 0.5).

**Table 2:** The extend of LD across the IL-13 and IL-4 genes

	SNP1	SNP2	SNP3	SNP4	SNP5
SNP1		0.5307	0.5333	0.3145	0.4065
SNP2			0.2953	0.1843	0.2393
SNP3				0.9728	0.9848
SNP4					0.9225
SNP5					

D' values among all single nucleotide polymorphism (SNP) pairs are shown. SNP1 is rs1800925, SNP2 is rs1295686, SNP3 is rs2227284, SNP4 is rs2243263, and SNP5 is rs2243267. LD, linkage disequilibrium.

As the two IL-13 SNPs are in different LD blocks than the three IL-4 SNPs, the haplotypes of the markers of the two genes were analyzed separately. Haplotype analysis of IL-13 predicted four haplotypes, all of which had a frequency above 1%. Compared with the most common haplotype CG, which is automatically selected as the reference haplotype, none of the haplotypes exhibited a significantly different distribution between cases and controls. For IL-4 a total of eight haplotypes were predicted, only three of which had a frequency above 1%. Again, no significant difference was detected between RA cases and healthy controls (Table 3).

**Table 3:** Haplotype frequencies and RA susceptibility

Haplotype	IL-13 haplotypes						IL-4 haplotypes						
	
	SNP1	SNP2	RA	Controls	OR (95% CI)	*P*	SNP3	SNP4	SNP5	RA	Controls	OR (95% CI)	*P*
1	C	G	0.754	0.747	1.00	-	C	G	G	0.760	0.766	1.00	-
2	T	A	0.110	0.107	1.02 (0.82–1.26)	0.86	A	G	C	0.136	0.128	0.89 (0.65 – 1.23)	0.49
3	C	A	0.065	0.078	0.83 (0.63–1.09)	0.19	A	C	G	0.090	0.091	1.38 (1.00 – 1.91)	0.96
4	T	G	0.068	0.067	1.02 (0.77–1.35)	0.91							

The frequency of haplotypes in each control is shown. SNP1 is rs1800925, SNP2 is rs1295686, SNP3 is rs2227284, SNP4 is rs2243263, and SNP5 is rs2243267. CI, confidence interval; OR, odd ratio; RA, rheumatoid arthritis; SNP, single nucleotide polymorphism.

### Single marker analysis with RA severity

We then examined the potential influence of these markers on disease severity, as defined by Modified Larsen scores and production of autoantibodies. No association was detected between the SNPs under study and markers of disease severity (Tables 4 and 5).

**Table 4:** Genotype frequencies of the genes involved in the IL-4/IL-13 pathway and modified Larsen score

Gene	SNP	Genotypes	Patients (*n*)	Median LS^a^	*P*
IL-13	rs1800925	TT	26	31.0	
		CT	271	27.0	0.8
		CC	608	28.0	
	rs1295686	TT	26	33.5	
		CT	251	25.0	0.8
		CC	580	28.0	
IL-4	rs2227284	AA	56	27.0	
		AC	316	29.0	0.6^b^
		CC	541	29.0	
	rs2243263	CC	8	41.5	
		AC	146	26.0	0.3
		AA	738	28.0	
	rs2243267	CC	18	23.5	
		CG	218	29.0	0.9
		GG	672	28.0	
IL-4R	rs1805010	GG	198	24.5	
		AG	460	30.0	0.5
		AA	250	29.0	
	rs1801275	AA	38	28.0	
		AG	316	27.5	0.5
		GG	558	29.0	

^a^Maximum possible modified (Larsen score) is 160. ^b^By Cuzick's test for trend. SNP, single nucleotide polymorphism.

**Table 5:** Genotype frequencies of IL-4/IL-13 variants and production of autoantibodies

Gene	SNP	Genotypes	Anti-CCP status^a^				RF status^a^			
			
			Positive (*n*)	Negative (*n*)	OR (95% CI)	*P*	Positive (*n*)	Negative (*n*)	OR (95% CI)	*P*
IL-13	rs1800925	TT	17	8	0.6 (0.3–1.8)	0.3^b^	16	7	1.0 (0.4–3.0)	1.0^b^
		CT	207	59	1.1 (0.7–1.5)	0.8	170	82	0.9 (0.7–1.3)	0.6
		CC	458	138	1.0		387	171	1.0	
	rs1295686	TT	20	6	1.0 (0.4–3.0)	1.0^b^	16	10	0.7 (0.3–1.8)	0.5^b^
		CT	192	54	1.0 (0.7–1.5)	0.8	161	72	1.0 (0.7–1.4)	1.0
		CC	439	128	1.0		367	165	1.0	
IL-4	rs2227284	AA	42	14	0.8 (0.4–1.7)	0.5	33	21	0.7 (0.4–14)	0.3
		AC	232	74	0.8 (0.6–1.2)	0.3	202	89	1.0 (0.8–1.5)	0.8
		CC	419	113	1.0		338	156	1.0	
	rs2243263	CC	5	3	0.5 (0.09–3.1)	0.4^b^	5	3	0.8 (0.1–5.0)	0.7^b^
		AC	102	38	0.8 (0.5–1.2)	0.2	89	44	0.9 (0.6–1.4)	0.2
		AA	568	160	1.0		465	213	1.0	
	rs2243267	CC	17	1	5.2 (0.8–218.9)	0.09^b^	13	4	1.5 (0.4–6.2)	0.6^b^
		CG	167	48	1.1 (0.7–1.6)	0.9	142	64	1.0 (0.7–1.4)	1.0
		GG	503	154	1.0		422	190	1.0	
IL-4R	rs1805010	GG	150	43	0.8 (0.5–1.4)	0.4	122	59	0.8 (0.5–1.3)	0.3
		AG	341	109	0.8 (0.5–1.2)	0.2	288	136	0.8 (0.6–1.2)	0.3
		AA	196	50	1.0		165	64	1.0	
	rs1801275	AA	31	5	2.0 (0.7–6.6)	0.2	26	9	1.4 (0.6–3.4)	0.6
		AG	241	71	1.1 (0.8–1.5)	0.7	197	92	1.0 (0.7–1.4)	1.0
		GG	416	131	1.0		351	165	1.0	

^a^The presence of anti-cyclic citrullinated peptide (anti-CCP) and rheumatoid factor (RF) was compared for each single nucleotide polymorphism (SNP) with reference to the commonest genotype. ^b^By Fisher's exact test for association. CI, confidence interval; OR, odds ratio.

### Gene-gene interactions between IL-4, IL-13 and IL-4R SNPs

The presence of epistatic interactions was initially examined by stratification analysis (Mantel-Haenszel test). Suggestive evidence of interactions was found when IL-4R Q551R was stratified by IL-13 -1112. The OR comparing individuals with the AA/AG genotypes at the IL-4R Q551R locus in participants who were TT at the IL-13 -1112 locus was higher than in those having either CC or CT genotypes (OR = 5.9 versus 1.0 in those who had the CC genotype and 1.3 in those having the CT genotype; *P *= 0.01 by Mantel-Haenszel test). However, these results are based on very small numbers and, after correction, the interaction is not statistically significant. No other interaction was detected.

## Discussion

In this study we conducted a haplotype-tagging approach to investigate the role played by the IL-4/IL-13 pathway in RA in a large case-control cohort. This approach allows us to examine the contribution of these genes with RA in a more robust manner than has previously been reported. Studies have reported the association of IL-4 and IL-4R with both RA susceptibility and severity [7,17,18], whereas the contribution of IL-13 variation has not previously been examined in RA. In our cohort single SNP analysis as well as haplotype analysis provided evidence of no association between RA development and variation in the IL-4/IL-13 pathway. Both genotype and allele frequencies were similar between cases and controls. Haplotype CGAGC yielded only a suggestive evidence for association, with a *P *value of 0.05. This haplotype contains the rare A allele of rs2227284 and the rare C allele of rs2243267, which are in high LD (D' = 0.98). Although none of this SNPs are known to be functional, the rs2243263 is in complete LD with the IL-4 -590 variant, which has been associated with differential promoter activity [5].

The IL-4R I50V variant has been associated with increased radiological damage during the first 2 years of disease [7], but we did not observe such an association. This conflicting finding could be due to differences in study design; our study is composed of a cross-sectional cohort with minimum disease duration of 3 years, whereas Prots and colleagues [7] conducted a prospective study with a follow up of 2 years. It is therefore possible that we missed some of the genetic effect in the first 3 years. Furthermore, the methods of assessing severity were different; we used Modified Larsen scores as a quantitative measure of structural damage, rather than comparison of genotypes between erosive and nonerosive RA.

Variants of IL-4 and IL-13 have been associated extensively with atopic asthma, serum concentrations of IgE and susceptibility to Crohn's disease [4,5,13,19-21]. Because these genes signal through a common pathway, gene-gene interactions may modify the effect of single SNPs in the IL-4/IL-13 pathway. Interaction analysis between IL-4/IL-13 and their shared receptor is of particular interest because gene-gene interactions may modify the effects of individual SNPs in the IL-4/IL-13 pathway, and evidence of epistasis has been reported in a asthma and type 1 diabetes [4,22,23]. Because the IL-4 and IL-13 genes are located on the same chromosome, we only investigated potential pair-wise interactions between each gene and IL-4R. Only suggestive evidence for epistasis was detected between IL-4R Q551R and IL-13 -1112, but this was not statistically significant after correction.

## Conclusion

We conclude that polymorphism in IL-4/IL-13/IL-4Ra loci do not contribute significantly in the genetic background of RA either individually or in combination.

## Abbreviations

CI = confidence interval; HWE = Hardy-Weinberg equilibrium; IL = interleukin; LD = linkage disequilibrium; OR = odds ratio; RA = rheumatoid arthritis; SNP = single nucleotide polymorphism; STAT = signal transducer and activator of transcription; TNF = tumour necrosis factor.

## Competing interests

The authors declare that they have no competing interests.

## Authors' contributions

AGW conceived and designed the study. All authors contributed to the acquisition of the samples or study data. IM performed genotyping as well as all data analyses. All the authors were involved in the interpretation of data. All the authors read and approved the final manuscript.
